# Analysis of the Effect of Thickness on the Performance of Polymeric Heart Valves

**DOI:** 10.3390/jfb14060309

**Published:** 2023-06-01

**Authors:** Jingyuan Zhou, Yijing Li, Tao Li, Xiaobao Tian, Yan Xiong, Yu Chen

**Affiliations:** 1 Department of Applied Mechanics, Sichuan University, Chengdu 610065, China; 2 College of Mechanical Engineering, Sichuan University, Chengdu 610065, China

**Keywords:** fluid–structure interaction (FSI), polymeric heart valves (PHVs), thickness, strain, stress

## Abstract

Polymeric heart valves (PHVs) are a promising and more affordable alternative to mechanical heart valves (MHVs) and bioprosthetic heart valves (BHVs). Materials with good durability and biocompatibility used for PHVs have always been the research focus in the field of prosthetic heart valves for many years, and leaflet thickness is a major design parameter for PHVs. The study aims to discuss the relationship between material properties and valve thickness, provided that the basic functions of PHVs are qualified. The fluid−structure interaction (FSI) approach was employed to obtain a more reliable solution of the effective orifice area (EOA), regurgitant fraction (RF), and stress and strain distribution of the valves with different thicknesses under three materials: Carbothane PC−3585A, xSIBS and SIBS−CNTs. This study demonstrates that the smaller elastic modulus of Carbothane PC−3585A allowed for a thicker valve (>0.3 mm) to be produced, while for materials with an elastic modulus higher than that of xSIBS (2.8 MPa), a thickness less than 0.2 mm would be a good attempt to meet the RF standard. What is more, when the elastic modulus is higher than 23.9 MPa, the thickness of the PHV is recommended to be 0.l–0.15 mm. Reducing the RF is one of the directions of PHV optimization in the future. Reducing the thickness and improving other design parameters are reliable means to reduce the RF for materials with high and low elastic modulus, respectively.

## 1. Introduction

Prosthetic heart valves are employed to replace the diseased native valve as a treatment for severe aortic valve (AV) disease. Mechanical heart valves (MHVs) and bioprosthetic heart valves (BHVs) are two main prostheses that have been utilized therapeutically; however, they are susceptible to thrombosis and structural valve degeneration (SVD), respectively [1,2]. It was found that the mechanical properties of polymer can easily be formulated to match that of native tissue and the geometry of it can be designed to produce physiological flow [3]. Consequently, polymeric heart valves (PHVs) are expected to overcome the shortcomings of MHVs and BHVs.

Materials with good durability and biocompatibility have always been the research focus in the field of PHVs for many years. Polysiloxanes, polytetrafluoroethylene (PTFE) and polyurethane (PUs) are the earliest materials applied for developing PHVs, which have good biocompatibility, hemodynamic properties and viscoelasticity, respectively [4,5]. Still, as time goes on, many PHVs made from them have been tested and failed because of thrombosis, calcification, hydrolysis, etc. [6]. Therefore, the challenges of durability remain. Novel polymers with better performance are obtained by adjusting the microstructure of materials with insufficient mechanical properties or reinforcing with filler. xSIBS and hydrogen−bonding−enhanced supramolecular hydrogels are typical cases of the former, and both of them have shown promising in vitro results [7,8,9]. Aerogel is widely used in medicine due to its attractive structural characteristics [10]. Macroscale composites have focused on reinforcing the elastomeric leaflets with macroscale fibers, such as Dacron (PET) and PTFE [11,12]. However, no commercially−viable devices have been developed using macroscopic composite materials so far. Nanocomposites have a better application foreground because of their superior mechanical properties, biocompatibility and simpler valve manufacturing process [3,4,13], such materials include polyhedral oligomeric silsesquioxane poly(carbonate–urea) urethane (POSS−PCU), polyvinyl alcohol (PVA) hydrogels reinforced with bacterial cellulose (BC) [14], the integration of graphene oxide (FGO) nanomaterials and PCUs [15], and the addition of carbon nanotubes (CNTs) to polymers with acceptable biocompatibility and mechanical properties [16,17]. Except for POSS−PCU that has undergone in vitro testing [18], the other three nanocomposites are still in the early stages of development.

The valve design is another crucial factor influencing the performance and lifetime of PHVs. Bending is a primary deformation mode of the leaflets. According to Euler−Bernoulli beam theory, bending stiffness is proportional to the cube of leaflet thickness [3]. Therefore, leaflet thickness is a major design parameter for PHVs. Previous studies qualitatively provided information on the relationship between leaflet modulus and thickness. The results show that the modulus and thickness are positively and negatively related to low stress, respectively [19]. Additionally, low modulus materials are subjected to higher strains than high modulus materials at the same stress level and are therefore more susceptible to creep and, ultimately, failure [20].

The performances of some PHVs made from novel materials have been tested in vitro hydrodynamic assessments. These include the Polynova xSIBS valve with variable thickness [7,8], the POSS−PCU valve with three leaflet thicknesses (0.1, 0.15 and 0.2 mm) [18], the PHV of silk fibroin fiber membranes (ISF) combined with poly (ethylene glycol) diacrylate (PEGDA) hydrogels with a thickness of 0.4 mm [21] and the PHV made from hyaluronan−enhanced linear−low−density polyethylene (LLDPE−HA) with a thickness of 0.08 mm [22], etc. What is more, the performances of PHVs are also well suited for measuring with the fluid–structure interaction (FSI) simulations due to the interplay between structure and fluid. The PHV constructed from bionate thermoplastic polycarbonate urethane with 0.127 mm thickness [23] and the PHV made from poly(styrene–ethylene–propylene–styrene) (SEPS) block copolymers with 0.20 mm thickness [24] were simulated where the flow rate was defined for the ventricular outflow rate. In addition, PHVs made from PU (0.16 mm) [25] and block co−polymer (0.3 mm) [26], as well as the Polynova xSIBS valve with variable thickness [7], were tested with the pressure boundary condition applied to the inlet section.

Due to the significant impact of the material and thickness of the PHV on its performance, it is necessary to adapt the thickness to the material to achieve optimal valve performance. However, whether it was testing the functionalities of PHVs made of novel polymers or focusing on improving design to enhance the performances of PHVs, few studies have mentioned how the thickness was selected. The former concerns mainly one material; therefore, its results have little reference significance for other materials. If the latter lacks consideration for thickness, the performance of the PHV obtained by optimizing other design parameters is probably not optimal.

The aim of the current work was to investigate the appropriate thickness range of PHVs that can meet basic functions. It is assumed that the appropriate thickness should enable PHVs to satisfy clinical requirements for the effective orifice area (EOA) and the regurgitant fraction (RF), as well as ensure that the stress and strain on the leaflets are small and evenly distributed. In this study, the FSI approach was employed for two cardiac cycles to obtain the performances of PHVs of three materials with different thicknesses. The selected materials consist of Carbothane PC−3585A, xSIBS and SIBS with higher molecular weight (Mn~70,000 g mol^−1^) reinforced by 1 wt% of CNTs (SIBS−CNTs). Carbothane PC−3585A is a kind of medical−grade thermoplastic polyurethane, it is widely used in the fields of biomedical applications [27]. xSIBS and SIBS−CNTs are the most promising developments in recent years [4,17]. SIBS−CNTs have enhanced biocompatibility and increased strength compared to neat SIBS [17], which will contribute to improving long−term durability and fatigue resistance, particularly in high−stress environments such as the cardiac cycle. The proposed thickness range supplies an option for the testing and optimization of PHVs with enhanced efficiency.

## 2. Materials and Methods

### 2.1. Geometry and Mesh Generation

The 3D geometry of the valve and blood flow volume were created in Rhino 7.0 (Robert McNeel & Assoc, Seattle, WA, USA). Both the structure and fluid were meshed using ANSA 21.0.1 (BETA CAE Systems, Root, Switzerland).

The geometry of the model was designed to resemble a typical prosthetic heart valve with leaflets in an almost closed configuration, based on the design of commercially available Sapien XT valve (Edwards Life Sciences, Irvine, CA, USA), setting the diameter to 23 mm and the height to 14.3 mm of all valves [28]. Leaflets were generated by the attachment curve, the free edge and the belly curve. The free edge was swept along the belly curve to intersect with the sinus at the attachment curve. We rotated one leaflet around the vertical axis to create three identical leaflets to generate the final valve model; each leaflet occupied 120° in the cross−section.

**Figure 1:**
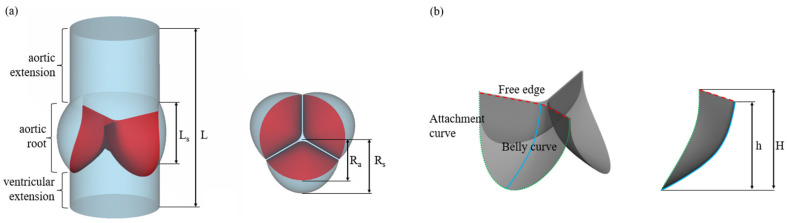
The geometry design of the model using the modeling design parameters. (**a**) Fluid field and (**b**) PHV.

The geometry of the aortic root in the current model was based on the measurements of aortic root in adults [29]. To move the boundary conditions away from the regions of interest, two straight−tube extensions with lengths of 10 and 20 mm were added to the upstream and downstream, respectively. The physiological significance of these lengths is that the upstream boundary was within the left ventricle, while the downstream boundary did not reach the aortic arch. Additionally, the two extensions have smaller influence on the results because the pressure drops in these extensions are obviously smaller than the transvalvular pressure difference of the valve [30]. The structural and fluid parts used for this study are shown in Figure 1. Dimension of the various design parameters are given in Table 1.

**Figure 2:**
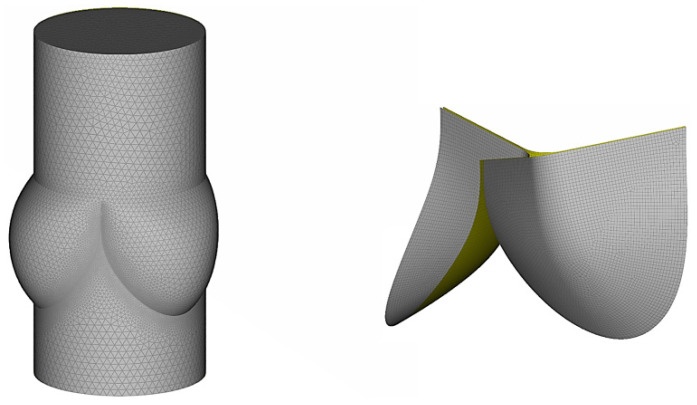
The discretization of fluid domain and PHV.

For spatial discretization, the valve was discretized into 17,130 quadrilateral fully integrated shell elements. Five integration points were distributed through shell thickness. The fluid domain was discretized into 529,152 tetrahedral Eulerian elements with maximum characteristic length of 1.0 mm (mean characteristic length of 0.60 mm). Finer resolution of 0.15 mm was used in the region surrounding the interface, which had the same geometry as the structure, and the attachment nodes of the interface were shared with the elements at the base of the sinus. The discretized solution fields for blood flow zone and valve leaflet structure are shown in Figure 2.

### 2.2. FSI Approach

FSI simulations with two−way coupling were applied to consider the interaction between the blood and PHVs. In order to limit the numerical instabilities caused by the approximate density of blood and PHVs, a strongly coupled strategy was implemented by the incompressible flow solver (ICFD) and the implicit mechanics solver in the LS−DYNA, Release 13.0 (Ansys, Canonsburg, PA, USA).

For FSI simulations, ICFD uses an Arbitrary Lagrangian−Eulerian (ALE) approach for mesh movement and models the interaction between the structural and fluid parts; the interfaces between the solid and the fluid are Lagrangian and deform with the structure. The solid and fluid geometry must match at the interface but not necessarily the meshes. Therefore, ICFD can automatically re−mesh to keep an acceptable mesh quality to support large deformation rate of the leaflets. Further, interaction detection coefficient (IDC, IDC = 0.25) was set to ensure FSI interaction.

### 2.3. Boundary Conditions

**Figure 3:**
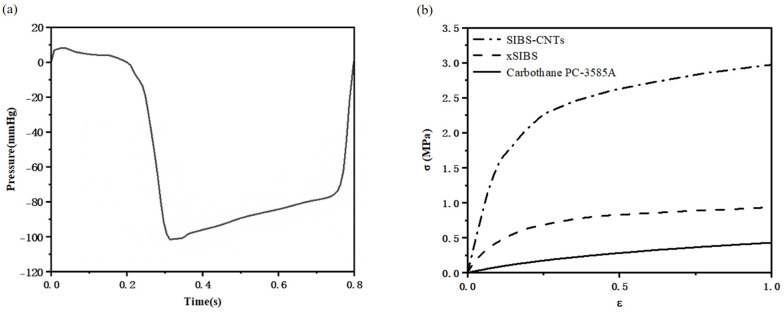
(**a**) The time−dependent physiological pressure difference between the left ventricle and the ascending aorta and (**b**) Engineering stress–strain curves of three materials.

The time−dependent physiological pressure difference between the left ventricle and the ascending aorta [31] was applied to the inlet (Figure 3a) and the outlet was set to zero pressure. A constant time step of 0.1 ms was set in the simulations. One cardiac cycle (0.8 s) was discretized in 8000 time steps. Two complete cardiac cycles were simulated to achieve convergence and eliminate the effect of sudden start during 1st cardiac cycle. All results were extracted from the 2nd cycle.

During the closing phase, the three leaflets experience significant contact with one another. Similar to previous studies, a penalty−based contact formulation and frictionless conditions were adopted here [32]. For two segments on each side of the contact interface that were overlapping and penetrating, a consistent nodal force assembly taking into account the individual shape functions of the segments was performed. When the three leaflets experienced significant contact with one another, both surface penetrations and edge−to−edge penetrations were checked.

For all models, the aortic wall boundaries were assumed rigid and no−slip condition was employed at the wall−blood interface. Surfaces of valve leaflets in contact with blood flow were considered as fluid−structure interfaces, across which loads and displacements were transferred. All nodes along the attachment curves were considered to be fixed in the FSI simulations.

### 2.4. Material Properties and Thickness of PHVs

The blood is assumed to be Newtonian and incompressible, with constant dynamic viscosity of 0.004 Pa∙s and a density of 1050 kg/m^3^ [33].

The selected polymers were modeled with 1st−order Ogden hyperelastic material models, which express the strain energy (*W*) by principal stretches ($\lambda_{1}$):

(1) $$W=\frac{\mu_{1}}{\alpha_{1}}\left(\lambda_{1}^{\alpha_{1}}-3\right)+\frac{1}{d_{1}}{\left(J-1\right)}^{2}\;$$

where $\lambda_{1}$ is the principal stretch ratio, $\mu_{1}$ and $\alpha_{1}$ are material constants, and $d_{1}$ is incompressible parameter. 

Based on the uniaxial tensile test results from previous research [17,34,35], the engineering stress–strain curves of the materials were used to solve material parameters of three materials according to the requirements in LS−DYNA [36]. Figure 3b shows the engineering stress–strain curves of three materials. The elastic modulus of xSIBS and SIBS−CNTs is 2.8 MPa and 23.9 MPa, respectively [17,37]. Because of the lack of data on the elastic modulus of Carbothane PC−3585A subjected to uniaxial tensile tests under the same conditions as [35], considering that the true stress–strain curve of Carbothane PC−3585A is almost linearly elastic within 40% strain, which is sufficient for PHV deformation, the elastic modulus of Carbothane PC−3585A is assumed to be 1.0 MPa in this study.

Taking the available application of Carbothane PC−3585A in PHV [35,38] into account, the thickness range of PHVs made from Carbothane PC−3585A was set to 0.25 mm, 0.30 mm and 0.35 mm. A variable thickness design has extensive applications in the investigations on PHVs made from xSIBS [7,34,37,39,40]. According to the design, the thickness range of PHVs made from xSIBS was set to 0.15 mm, 0.20 mm and 0.25 mm. Materials with an elastic modulus greater than 20 MPa, similar to SIBS−CNTs, were used to produce PHVs with a thickness of less than 0.15 mm for testing [18,23], while too small thickness may cause the PHV to prolapse [19]. Therefore, the thickness of SIBS−CNTs was set to 0.10 mm, 0.15 mm and 0.2 mm.

## 3. Results

### 3.1. Valve Performance Parameters

In order to assess the quantification of valve stenosis, the effective orifice area (EOA) was used as a measurement of the effective jet area during left ventricular ejection of the cardiac cycle [41]. The EOA was calculated using Equation (2) [42]:

(2) $$\mathrm{EOA}=\frac{q_{vRMS}}{51.6\sqrt{\frac{\Delta p}{\rho }}}\;$$

where $q_{vRMS}\;\left({\mathrm{cm}}^{3}/s\right)$ is the root mean square of the forward flow rate during the positive differential pressure period; $\Delta p\;\left(\mathrm{mmHg}\right)$ is the average pressure difference measured during the positive differential pressure period; and $\rho \;\left(g/{\mathrm{cm}}^{3}\right)$ is the density of the fluid. According to the boundary conditions shown in Figure 3a, Δp=4.79 mmHg, which was close to that in the healthy young condition [42].

Regurgitant fraction (RF) evaluates leakage after valve closure; Equation (3) from ISO 5840−3:2021 [43] was applied to calculate the RF:

(3) $$RF=\frac{V_{C}+V_{L}}{V_{F}}\times 100\%$$

where *V_c_* is the closing volume, *V_L_* is the leakage volume and *V_F_* is the forward volume. 

**Figure 4:**
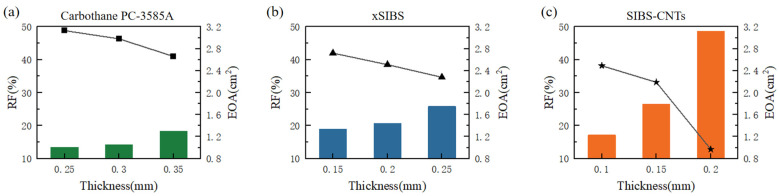
EOA (bars, left Y−axis units) and RF (points and connecting lines, right Y−axis units) of (**a**) Carbothane PC−3585A, (**b**) xSIBS and (**c**) SIBS−CNTs.

The EOA and RF calculated by Equations (2) and (3), respectively, are shown in Figure 4. PHVs made from Carbothane PC−3585A obtained larger EOA and smaller RF. The EOA and RF of the thinnest PHV (0.15 mm) of xSIBS were equivalent to those of the thickest PHV (0.35 mm) made from Carbothane PC−3585A. More significant changes were observed in the results of SIBS−CNTs. The PHV made from SIBS−CNTs with a thickness of 0.20 mm had the smallest $V_{F}$ (21.31 mL), as shown in Table 2. As the thickness reduced to 0.15 mm, the $V_{F}$ rapidly increased to 52.39 mL; still, large regurgitation made its RF relatively high. In addition, satisfactory results were achieved when the thickness was reduced to 0.10 mm.

### 3.2. Valve Dynamics

**Figure 5:**
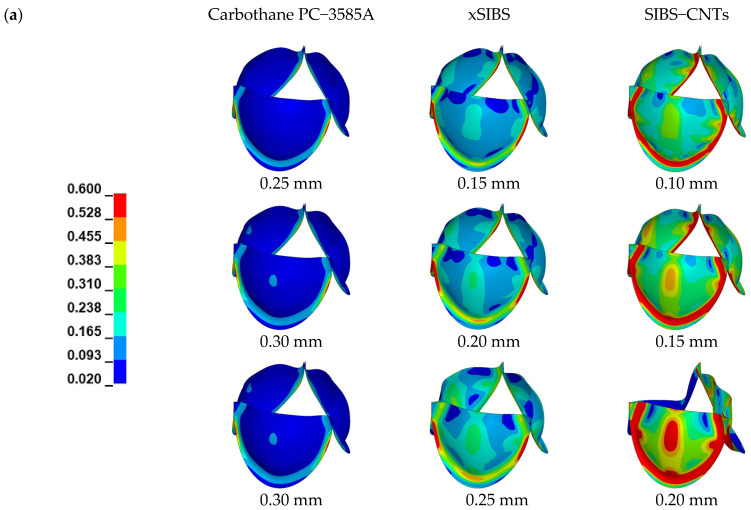

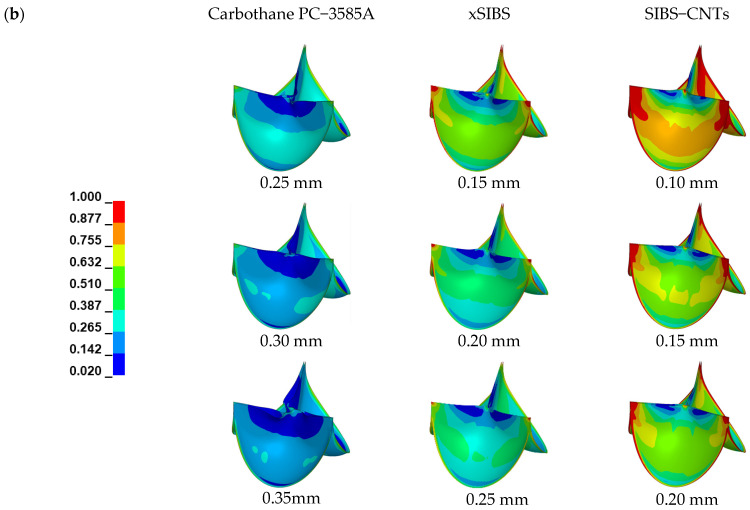
von−Mises stresses (MPa) distribution of the three material models. (**a**) At the moment of fully opening and (**b**) at the moment of fully closing. Note that the color scales are different for fully−opened and fully−closed results.

**Figure 6:**
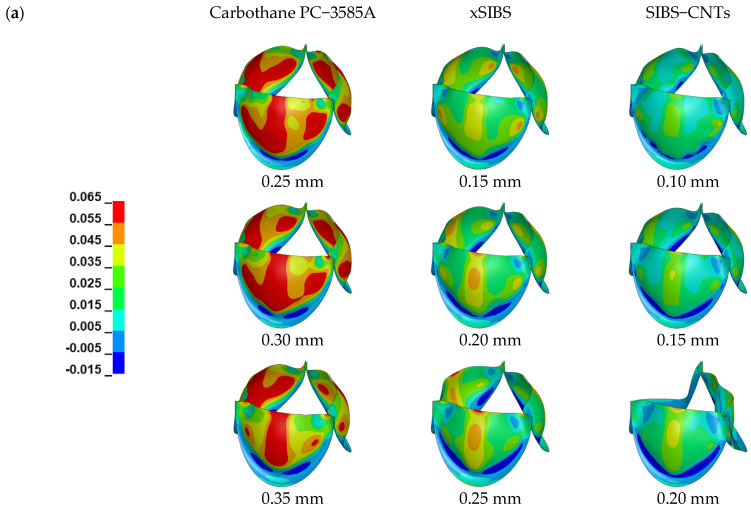

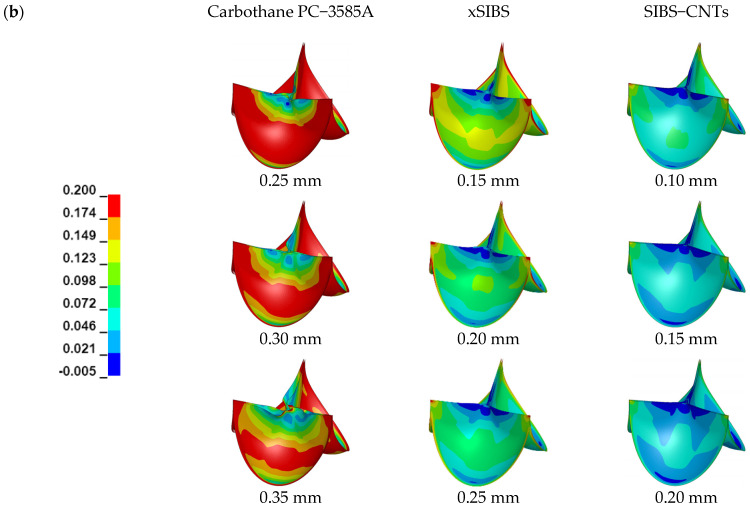
MIPE distributions of the three material models. (**a**) At the moment of fully opening and (**b**) at the moment of fully closing. Note that the color scales are different for fully−opened and fully−closed results.

Figure 5 shows von Mises stresses distribution on the leaflets. The von Mises stresses were used to quantify the stress field in the leaflets because the yield behavior of polymers is often described by modified versions of the von Mises criterion [44]. Similar to previous analyses [45,46,47], the maximum in−plane principal Green−Lagrange strain (MIPE) on the aortic side of the leaflets, as shown in Figure 6, was applied to include contributions from both stretching and bending [45].

Figure 5 depicts that the stress concentrations distributed along the attachment curve and at commissure points were negatively correlated with the thickness. The valves made from Carbothane PC−3585A had the lowest stresses during the entire cardiac cycle. The valve (0.10 mm) made from SIBS−CNTs had the maximum stress value of all the models, which was 2.15 MPa after the valves were completely closed.

Figure 6 shows that the valves made from Carbothane PC−3585A had strains significantly higher than the valves made from other materials, especially at the fully−closed stage. The same distribution of strains was observed for xSIBS and SIBS−CNTs. That is, at the fully−opened stage, as thickness increased, the strain in the middle of the valve belly became more obvious and the high strain on both sides of the belly came to disappear; at the same time, the low strain area at the bottom of the belly expanded. After the valve was fully closed, high strains along the attachment curve and at commissure points were reduced with the increase in thickness, which also made the strain distribution on the valve more uniform.

## 4. Discussion

With the same PHV design and boundary conditions, it can be observed from Figure 4 that EOA and RF were negatively and positively related to the thickness, respectively, with the same material, this result ties well with the test results of POSS−PCU [18] that are shown in Table 3 and it is more obvious with the increase in elastic modulus. According to the performance requirements for PHVs with a diameter of 23 mm in ISO 5840−3:2021: RF < 20%, EOA > 1.25 cm^2^. PHVs made from materials that have a smaller elastic modulus (<2.8 MPa) can achieve the EOA and RF that met the standard within the thickness of 0.15–0.20 mm. This is especially true for materials with a higher elastic modulus, otherwise it will be difficult to obtain qualified performance parameters, such as the PHV of SEPS [48], which had a smaller geometric orifice area (the GOA shown in Table 3) under the same flow rate of 4 L/min as the xSIBS−PHV (0.25 mm) calculated in this study. 

The thickness of 0.20 mm seriously hampered the complete opening of the PHV for SIBS−CNTs with an elastic modulus of 23.9 MPa. Fortunately, the decrease in thickness rapidly increased the CO, significantly enlarged EOA and reduced RF. However, it should be noted that reducing the thickness to improve the performance of PHVs may be more suitable for materials with a high elastic modulus, as they have better resistance to the leaflet flutter caused by reduced thickness [49]. On balance, materials with an elastic modulus higher than 23.9 MPa can be used to make PHVs with a thickness of less than 0.15 mm to achieve satisfactory EOA and RF.

Compared to previous studies, the RF in this study seems to be higher. On the one hand, the same physiological pressure pulse was applied as the pressure boundary condition for all simulations. The greater the elastic modulus of the material, the greater the pressure differential that should be applied to ensure the complete closure of the valve [19]. Therefore, the high RF shown in Figure 4 can indicate that the normal physiological pressure difference is not sufficient to completely close PHVs of xSIBS with a thickness of 0.25 mm and PHVs of SIBS−CNTs with a thickness greater than 0.15 mm. PEGDA−ISF demonstrated a similar situation, it had a bigger elastic modulus (4.54 ± 0.43 MPa) than xSIBS while the PHV (0.4 mm) made from PEGDA−ISF showed a qualified RF (14.5%), this may be caused by the larger pressure difference (Figure 5c of [21]) in the diastole of the experiment than the 100 mmHg used here. On the other hand, in vitro observations of PHVs made from xSIBS indicate that the RF was decreased during long−term experiments [7], while it is difficult for FSI to observe such an obvious phenomenon in multiple cycles because of its high calculation cost [50].

Although higher stresses were observed from the valves made from xSIBS and SIBS−CNTs, the thicknesses set for them were smaller than the thickness range of Carbothane PC−3585A; hence, it is reasonable to observe greater stresses by the joint effect of thinner thicknesses and a bigger elastic modulus. 

A qualified EOA and RF can be obtained easier in the valve made from Carbothane PC−3585A even if the thickness of the valve was large, however, the strain of such valves was significantly higher; the strain accumulation due to high strain is related to creep and valve failure [20,49]. This is consistent with previous finite element results, that is, the strain of the valve increases as the elastic modulus of its material decreases [19]. However, it should be noted that strains changed obviously with the elastic material. At the fully−closed stage, it is observed from Figure 6 that the maximum strain of valves decreased from 40.1% to 20.6% only by raising the elastic modulus from 1 MPa (Carbothane PC−3585A) to 2.8 MPa (xSIBS) at the same valve thickness (0.25 mm). Smaller and uniformly distributed strains can be observed in SIBS−CNTs; even in the thinnest PHV (0.1 mm), its maximum strain did not exceed 20%.

**Figure 7:**
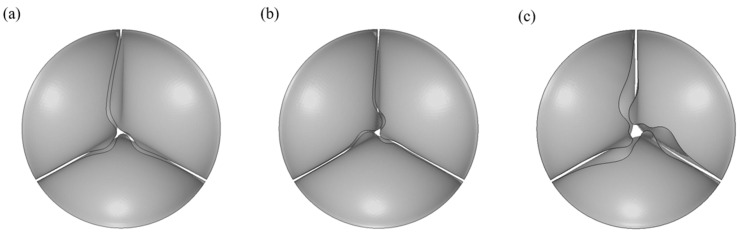
The top view of PHVs of Carbothane PC−3585A after the PHVs were completely closed. (**a**) 0.25 mm, (**b**) 0.30 mm and (**c**) 0.35 mm.

In addition, the results show that the design of the PHV is also a key element in valve performance. On the one hand, after the closure of the valve (0.35 mm) made from Carbothane PC−3585A, the observed unnecessary distortion from Figure 7 indicated that there is a relationship between thickness and the length of the free edge. In this case, reducing the length of the free edge appropriately may allow more complete closure, thereby reducing the regurgitation. On the other hand, the RF of valves made of xSIBS (Figure 4) is higher than that of the PolyNova Valve, a novel xSIBS polymer−based valve which has a special design as shown in [7]. Apart from changing the radial cross−sectional profile of the leaflets from uniform to variable thickness, the coaptation height is equal to the valve height. Previous literature suggests that decreasing the ratio of valve height to coaptation height increases the coaptation area (CA) of the leaflets significantly, and a large CA reduces the possibility of aortic regurgitation [51]. Therefore, the design of the PolyNova Valve may explain why the RF obtained through in vitro experiment [7] is lower than the RF shown in Figure 4. 

**Table 3:** Available data on material properties and performance assessment of PHVs in recent years.

Material	Elastic Modulus (E)	Thickness	Test Standard	Results	References
POSS−PCU	E = 26.2 MPa	0.10 mm	In vitro hydrodynamic assessment: ISO 5840−3	EOA = 3.34 cm^2^, RF = 4.68%	[18]
	E = 23.0 MPa	0.15 mm		EOA = 3.13 cm^2^, RF = 10.77%	
	E = 15.9 MPa	0.20 mm		EOA = 2.69 cm^2^, RF = 12.34% (10 cycles)	
xSIBS	E = 2.8 MPa	Various thicknesses (0.15–0.25 mm)	In vitro hydrodynamic assessment: ISO 5840−3	EOA = 1.8 ± 0.04 cm^2^, RF = 7.5 ± 1.0% (400 million cycles)	[7]
				EOA = 1.7 cm^2^, RF = 16.7% (10 cycles)	[8]
PEGDA−ISF	E = 4.54 ± 0.43 MPa	0.4 mm	In vitro hydrodynamic assessment: ISO 5840−3	EOA = 2.30 cm^2^, RF = 14.5% (10 cycles)	[21]
LLDPE−HA *	E > 76.49 ± 1.86 MPa	0.08 mm	In vitro hydrodynamic assessment: a pulsatile flow driving the PHV, 1 s is one cycle.	EOA= 2.08 ± 0.04 cm^2^, RF= 11.23 ± 0.55 (60 cycles)	[22]
PU	E = 3.67 MPa	0.15 mm	In vitro hydrodynamic assessment: a pulsatile flow driving the PHV, 1 s is one cycle.	GOA = 3.90 cm^2^ (24 cycles)	[50]
	E = 8 MPa	0.16 mm	FSI: Pressure difference (600 bpm) (inlet)	GOA = 2.83 cm^2^ (3 cycles)	[25]
Bionate thermoplastic polycarbonate urethane	E = 23.93 MPa	0.127 mm	FSI: low rate that was equal to 4.5 L/min of CO (inlet) and a mean arterial pressure of 100 mmHg (outlet)	GOA = 3.31 cm^2^, RF = 5.64% (4 cycles)	[23]
SEPS	E = 3.2 MPa	0.20 mm	FSI: flow rate (inlet) and zero pressure (outlet)	GOA = 2.24 cm^2^ (0.4 s)	[24]
		Two leaflets with a thickness of 0.36 mm and one leaflet of 0.39 mm	FSI: pressure difference corresponded to a flow rate of 4 L/min	GOA = 1.81 cm^2^ (2 cycles)	[48]

* The description of HA content is vague in [22], but the addition of HA will cause the elastic modulus of LLDPE to be at least higher than 76.49 ± 1.86 MPa according to [52].

### Limitation

This paper solely discussed the relationship between thickness and the elastic modulus of materials based on TAVI Sapien−XT leaflet design. The impact of other designs on the performance of the valve has not been taken into account. From the results, it can be seen that the TAVI Sapien−XT leaflet design used in this study was not entirely suitable for materials with high elastic modulus. Although how to improve the design for such materials has been discussed, and the conclusions were obtained by comparing the results with other studies shown in Table 3, which should reduce the impact of a single design to some extent, the limitations brought about by the design need to be further pointed out.

Furthermore, due to the limited scope of this work, further tests must be performed in vitro and in vivo with PHVs to assess how the thickness affects the long−term durability of the device. For example, PHVs have shown excellent potential in TAVR procedures [7,8,16,53], which requires the valve to be crimped at a low diameter for the purpose of catheter insertion [54]. It is obvious that the process is closely related to the material and thickness of the PHVs. Therefore, it is necessary to conduct crimping experiments on the PHVs to evaluate the effectiveness and stability of the material under high crimping strain conditions.

Finally, isotropic nonlinear hyperelastic material was assumed for the mechanical properties of the leaflets, more comprehensive results can be obtained by considering anisotropic materials.

## 5. Conclusions

This study investigated the relationship between valve thickness and material properties using FSI simulations. Three polymer materials, Carbothane PC−3585A, XSIBS and SIBS−CNTs were selected. The smaller elastic modulus of Carbothane PC−3585A allowed for a thicker valve (>0.3 mm) to be produced, while for materials with an elastic modulus higher than that of xSIBS (2.8 MPa), a thickness less than 0.2 mm would be a good attempt to meet the RF standard. What is more, when the elastic modulus is higher than 23.9 MPa, the recommended thickness of the PHV is 0.l−0.15 mm. Reducing the RF is one of the directions of PHV optimization in the future. Materials with a high elastic modulus can resist high strain, so it is advisable to reduce backflow by decreasing the thickness. However, considering that a reduction in the elastic modulus of the material will significantly increase the strain on the PHV, for materials with a small elastic modulus, adjusting other design optimization parameters based on an appropriate thickness is more secure.

## Figures and Tables

**Table 1:** Dimensions of design parameters used in modeling the AV.

	H (mm)		h (mm)	
Structure	14.3		11.3	
	R_a_ (mm)	R_s_ (mm)	L_s_ (mm)	L (mm)
Fluid	11.5	15.0	17.0	47.0

H: valve height, h: leaflet coaptation height, R_a_: radius of aorta, R_s_: radius of sinus, L_s_: sinus length, L: model length.

**Table 2:** The cardiac output (CO) and regurgitation of all models.

	Carbothane PC−3585A			xSIBS			SIBS−CNTs		
*t* (mm)	0.25	0.30	0.35	0.15	0.20	0.25	0.10	0.15	0.20
$V_{c}+V_{L}$ (mL)	6.81	10.75	12.32	12.94	12.76	14.33	10.46	13.89	10.36
$V_{F}$ (mL)	80.34	76.03	67.01	68.23	61.72	55.49	61.00	52.39	21.31

## Data Availability

Not applicable.
